# Gastrites chroniques à hélicobacter *pylori*: évaluation des systèmes OLGA et OLGIM

**DOI:** 10.11604/pamj.2016.23.28.8839

**Published:** 2016-02-04

**Authors:** Sana Ben Slama, Dorra Ben Ghachem, Amen Dhaoui, Mohamed Taieb Jomni, Mohamed Hédi Dougui, Khadija Bellil

**Affiliations:** 1 Service d'Anatomie Pathologique, Hôpital M. Slim La Marsa, Tunisie; 2 Service d'Anatomie Pathologique, Hôpital des FSI La Marsa, Tunisie; 3 Service de Gastro-entérologie, Hôpital des FSI La Marsa, Tunisie

**Keywords:** Gastrite chronique, helicobacter pylori, atrophie gastrique, métaplasie intestinale, classification, Chronic gastritis, helicobacter pylori, atrophic gastritis, intestinal metaplasia, staging

## Abstract

La gastrite chronique à *Helicobacter pylori (H pylori)* présente un risque de cancérisation en rapport avec l'atrophie et la métaplasie intestinale. Deux nouvelles classifications, OLGA (Operative Link on Gastritis Assessment) et OLGIM (Operative Link on Gastritic Intestinal Metaplasia assessment) ont été proposées pour individualiser les formes à haut risque évolutif (stades III et IV). Le but de ce travail est d’évaluer les classifications de OLGA et de OLGIM au cours des gastrites chroniques à *H pylori*. Nous avons réalisé une étude descriptive transversale portant sur 100 cas de gastrite chronique à *H pylori*. La réévaluation des paramètres du Sydney System de l'atrophie et de la métaplasie intestinale, de l'antre et du corps gastrique, a permis de définir respectivement les stades OLGA et OLGIM. Le risque évolutif de nos gastrites à *H pylori* était de 6% selon OLGA et de 7% selon OLGIM. Une liaison significative a été révélée entre l’âge et OLGA. Les gastrites à haut risque selon OLGIM, étaient significativement associées à une atrophie modérée à sévère. Les formes à haut risque selon OLGA s'associaient dans plus de 80% des cas à une métaplasie intestinale. OLGA et OLGIM présentaient une corrélation positive et hautement significative entre elles avec une discordance évaluée à 5%. Les classifications de OLGA et OLGIM, en complément au Sydney System, permettent de sélectionner les formes de gastrites à haut risque nécessitant une surveillance étroite.

## Abstract (en)

Helicobacter pylori (H pylori) gastritis presents a risk of cancer related to atrophy and intestinal metaplasia. Two recent classifications OLGA (Operative Link on Gastritis Assessment) and OLGIM (Operative Link on Gastritic Intestinal Metaplasia assessment) have been proposed to identify high-risk forms (stages III and IV). The aim of this study is to evaluate the OLGA and OLGIM staging systems in H pylori gastritis. A descriptive study of 100 cases of chronic H pylori gastritis was performed. The revaluation of Sydney System parameters of atrophy and intestinal metaplasia, of gastric antrum and corpus, allowed identifying respectively the stages of OLGA and OLGIM systems. The progressive risk of our H pylori gastritis was 6% according to OLGA staging and 7% according to OLGIM staging. Significant correlation was revealed between age and OLGA staging. High-risk gastritis according to OLGIM staging was significantly associated with moderate to severe atrophy. High-risk forms according to OLGA staging were associated in 80% of the cases to intestinal metaplasia. OLGA and OLGIM systems showed a highly significant positive correlation between them with a mismatch at 5% for H pylori gastritis. The OLGA and OLGIM staging systems in addition to Sydney System, allow selection of high risk forms of chronic gastritis requiring accurate observation.

## Introduction

La gastrite chronique englobe un groupe d'affections le plus souvent d'origine infectieuse liée à *Helicobacter pylori (H pylori)*, ou plus rarement d'origine auto-immune. Elle se définie histologiquement comme un état inflammatoire persistant de la muqueuse gastrique diffus ou localisé, associé à des altérations épithéliales pouvant évoluer vers l'atrophie et/ou la métaplasie intestinale (MI) [1]. En pratique, la gastrite chronique soulève une double problématique liée d'une part à son risque de dégénérescence carcinomateuse et d'autre part, à l'existence de difficultés de classification et de sélection des patients nécessitant une surveillance particulière. La gastrite chronique a fait l'objet de multiples classifications. Le Sydney System est le plus communément utilisé en pratique courante [1]. Il permet une graduation claire des lésions précancéreuses. Il a l'inconvénient de ne pouvoir sélectionner les formes de gastrites chroniques à haut risque évolutif qui nécessiteraient une surveillance particulière. C'est dans ce contexte que deux nouvelles classifications des gastrites chroniques définies par: « Operative Link on Gastritis Assessment» (OLGA) [2] et « Operative Link on Gastritic Intestinal Metaplasia assesment » (OLGIM) [3] étaient proposées et permettent d’établir un score global d’évolutivité en fonction des degrés d'atrophie et de MI. Dans notre étude, nous nous proposons d’évaluer les classifications OLGA et OLGIM sur 100 cas de gastrite chronique à *H pylori* et de déterminer les éventuelles corrélations entre les classifications de OLGA et de OLGIM, et les données épidémiologiques et anatomopathologiques.

## Méthodes

*Type d’étude:* c'est une étude rétrospective transversale portant sur 100 cas de gastrite chronique à *H pylori* colligés au service d'anatomie pathologique de l'hôpital des FSI (forces de sécurité de l'intérieur) de la Marsa (Nord de Tunis) sur une période d'un an (janvier-décembre 2012).

*Critères d'inclusion:* nous avons inclus les cas de gastrite chronique à *H pylori*, diagnostiquées sur biopsies gastriques.

*Critères de non inclusion:* Les biopsies non incluses dans notre étude sont celles portant sur les formes de gastrites sans signes d'infection à *H pylori*.

*Critères d'exclusion:* nous avons exclu les gastrites chroniques à *H pylori* concernant les patients dont l’âge est inférieur à 15 ans, ayant reçu un traitement par les inhibiteurs de la pompe à protons (IPP), les gastrites à *H pylori* au voisinage d'une néoplasie maligne ou d'un ulcère duodénal, les gastrites à *H pylori* associées à une autre pathologie notamment de reflux ou gastrites avec signes évocateurs d'une origine auto-immune, les gastrites granulomateuses à *H pylori*, et les prélèvements biopsiques de mauvaise qualité ou non représentatifs du corps gastrique.

*Le recueil des données:* épidémiologiques (âge, sexe), endoscopiques (la topographie des lésions) et anatomopathologiques a été effectué pour l'ensemble de nos 100 patients. Les prélèvements ont porté sur des échantillons de muqueuses antrale, de l'angle de la petite courbure et du corps gastrique (fundique).

*Échantillons tissulaires:* tous les prélèvements étaient fixés dans du formol tamponné à 10%. Les biopsies étaient traitées selon une technique conventionnelle, et analysées après coloration usuelle à l'hématoxyline-éosine (HE) et spéciale au bleu alcian (pH 2.5) pour la recherche de mucines acides et au Giemsa modifiée pour la mise en évidence de l’*H pylori*.

*Paramètres histologiques étudiés:* dans un premier temps, on a appliqué la classification du Sydney system pour toutes nos lames en évaluant les paramètres suivants : Inflammation, Activité, Atrophie et MI, cotés en absente, légère, modérée à sévère et d'autres paramètres non gradables (autres altérations épithéliales, hyperplasie neuroendocrine…). Et finalement, la recherche et de l’*H pylori* sur l'HE coloration de Giemsa modifiée: *H pylori* apparaissait sous forme de bacilles incurvés ou en « S » de 3 à 4 µm de largeur, fortement basophile sur coloration de Giemsa modifiée. La densité était estimée à légère, modérée ou sévère. Ceci nous a permis d'individualiser le groupe de gastrite à *H pylori* (100 cas). Puis, on y a appliqué les classifications de OLGA et de OLGIM.

La classification OLGA: se base sur l’évaluation semi-quantitative de l'intensité des lésions atrophiques des muqueuses fundique et antrale avec un staging allant, de 0 à IV. Les stades 0, I et II représentent des stades à faible risque évolutif. Les stades III et IV définissent les stades à haut risque évolutif (Tableau 1). La classification OLGIM: consistait à subdiviser les patients en cinq classes (de 0 à IV) selon le grade et le siège de la MI définissant ainsi le score global de la métaplasie intestinale (Tableau 2). Pour ces deux classifications, les stades 0, I et II représentent des stades à faible risque évolutif. Les stades III et IV définissent les stades à haut risque évolutif.

**Tableau 1:** Classification OLGA, d'après Rugge et al [2]

Score de l'atrophie		CORPS GASTRIQUE			
		Absente	Légère (glandes atrophiques <30%)	Modérée (glandes atrophiques 30%-60%)	Sévère (glandes atrophiques >60%)
ANTRE	Absente	Classe 0	Classe I	Classe II	Classe II
	Légère (glandes atrophiques <30%)	Classe I	Classe I	Classe II	Classe III
	Modérée (glandes atrophiques 30%-60%)	Classe II	Classe II	Classe III	Classe IV
	Sévère (glandes atrophiques >60%)	Classe III	Classe III	Classe IV	Classe IV

**Tableau 2:** Classification OLGIM, d'après Capelle et al. [3]

Score de la métaplasie intestinale		CORPS GASTRIQUE			
		Absente	Légère (glandes en MI <30%)	Modérée (glandes en MI 30%-60%)	Sévère (glandes en MI >60%)
ANTRE	Absente	Classe 0	Classe I	Classe II	Classe II
	Légère (glandes en MI <30%)	Classe I	Classe I	Classe II	Classe III
	Modérée (glandes en MI 30%-60%)	Classe II	Classe II	Classe III	Classe IV
	Sévère (glandes en MI >60%)	Classe III	Classe III	Classe IV	Classe IV

*Étude statistique:* les analyses statistiques étaient effectuées à l'aide du logiciel de statistiques SPSS 13 ®. Une description globale de l'ensemble de l’échantillon était réalisée en donnant les fréquences des différentes catégories pour les variables qualitatives. Pour étudier la liaison entre deux variables, il a été fait appel au test de du Chi-deux. Les différences ont été considérées comme significatives lorsque p était inférieur à 0,05.

## Résultats

### Données épidémiologiques

Notre étude a porté sur 100 cas de gastrites chroniques à *H pylori*. La répartition de nos cas selon les différents stades des classifications OLGA et OLGIM ainsi que les données épidémiologiques sont présentées dans le Tableau 3. Concernant la classification OLGA, les stades à faible risque évolutif (0, I et II) représentaient 94% des cas et ceux à haut risque évolutif (III et IV), 6%. Quant à la classification OLGIM, les stades à faible risque évolutif représentent 93% des cas et ceux à haut risque évolutif 7%. L’âge moyen de nos patients était de 42,73 ans (avec des extrêmes allant de 16 à 75 ans), un pic de fréquence était observé entre 40 et 60 ans. Il y avait 41 hommes et 59 femmes soit un sex ratio H/F de 0,69. L’étude comparative montrait une corrélation positive (c=0,29) et statistiquement significative entre l’âge et la classification OLGA (p=0,03) et l'absence de liaison entre le sexe et la classification OLGA (p=0,9). Il n'y avait pas de corrélation entre la classification OLGIM et l’âge (p=0,06) et entre la classification OLGIM et le sexe (p=0,48).

**Tableau 3:** Répartition des données épidémiologiques et de la densité de *H pylori* selon les différents stades des classifications OLGA et OLGIM

	Stade 0		Stade I		Stade II		Stade III		Stade IV	
	OLGA	OLGIM	OLGA	OLGIM	OLGA	OLGIM	OLGA	OLGIM	OLGA	OLGIM
**Nombre de Cas** (%)	24 (24)	49 (49)	62 (62)	40 (40)	8 (8)	4 (4)	5 (5)	5 (5)	1 (1)	2 (2)
**Age Moyen** (ans)	35,7	41,3	43,5	45,2	54,6	39	44,2	35,2	58	53
**Sexe** (H/F)	9/15	22/27	27/35	13/27	3/5	3/1	2/3	2/3	0/1	1/1
**Densité** ***H pylori*** (cas)	24	49	62	40	8	4	5	5	1	2
Légère (cas/%)	7/29	19/39	23/37	13/32	2/25	0	3/60	2/40	1/100	2/100
Modérée (cas/%)	7/29	12/24	18/29	12/30	1/13	2/50	0	1/20	0	0
Sévère (cas/%)	10/42	18/37	21/34	15/38	5/62	2/50	2/40	2/40	0	0

### Densité en *H pylori*

La densité en *H pylori* était légère dans 37 cas (37%), modérée dans 26 cas (26%) et sévère dans 37 cas. Il n'y avait pas de différence significative entre les stades OLGA et OLGIM en fonction de la densité en *H pylori* (respectivement p=0,32 et p=0,82). En divisant nos gastrites en deux groupes; le premier à faible densité en *H pylori* (densité légère) et le deuxième à forte densité en *H pylori* (modérée à sévère), on obtient pour la classification OLGA, des gastrites à faible risque significativement associées à une forte densité en *H pylori* (p<0,05); estimée à 71% pour le stade 0, 63% pour le stade I et 75% pour le stade II. Les gastrites à haut risque étaient significativement associées à une faible densité en *H pylori* (p<0,05); estimée à quatre cas sur six. Concernant la classification OLGIM, les gastrites à faible risque étaient significativement associées à une forte densité en *H pylori* (p <0,05); estimée à 61% pour le stade et 68% pour le stade I et 100% pour le stade II. Les gastrites à haut risque étaient significativement associées à une faible densité en *H pylori* (p<0,05); estimée à quatre cas sur sept.

### Atrophie glandulaire

Parmi nos 100 cas, l'atrophie était présente dans 76 cas (76%). L'atrophie était légère dans 62 cas (62%), modérée dans 13 cas (13%) et sévère dans un cas (1%). La répartition était différente selon le siège. En effet, au niveau du fundus, l'atrophie était présente dans 42 cas (54%), légère dans 35 cas (45%), modérée dans six cas (8%) et sévère dans un cas (1%). Au niveau de l'antre, l'atrophie retrouvée dans 62 cas (62%) était estimée à légère dans 59 cas (59%), modérée dans 12 cas (12%) et sévère dans un cas (1%). L'atrophie antrale était en corrélation statistique très significative (p<0,005) avec l'atrophie fundique. La répartition des stades de OLGIM selon l'intensité de l'atrophie est résumée dans le Tableau 4. L’étude comparative montrait une différence très significative entre les stades OLGIM en fonction de l'intensité de l'atrophie (p<0,005). En effet, pour les gastrites chroniques de faible risque selon OLGIM, l'atrophie était présente avec une intensité le plus souvent légère (64%). Nos sept cas de gastrites à haut risque, étaient associées à une atrophie glandulaire d'intensité « modérée à sévère » dans six cas/sept.

**Tableau 4:** Répartition des stades de OLGIM selon l'intensité de l'atrophie et des stades de OLGA selon l'intensité de la métaplasie intestinale

	Stade 0	Stade I	Stade II	Stade III	Stade IV
**Atrophie dans OLGIM**					
Absente (cas/%)	17 (35)	6 (15)	1 (25)	0	0
Légère (cas/%)	31 (63)	26 (65)	1 (25)	1 (20)	0
Modérée (cas/%)	1 (2)	8 (20)	1 (25)	2 (40)	1 (50)
Sévère (cas/%)	0	0	1 (25)	2 (40)	1 (50)
**Métaplasie intestinale dans OLGA**					
Absente (cas/%)	17 (71)	31 (50)	0	1 (20)	0
Légère (cas/%)	6 (25)	26 (42)	0	1 (20)	0
Modérée (cas/%)	0	5 (8)	1 (13)	2 (40)	0
Sévère (cas/%)	1 (4)	0	0	1 (20)	1 (100)

### Métaplasie intestinale (MI)

Parmi nos 100 cas, la MI était retrouvée dans 49 cas (49%), elle était légère dans 40 cas (40%), modérée dans six cas (6%) et sévère dans trois cas (3%). Au niveau du fundus, La MI retrouvée dans 27 cas, était légère dans 20 cas, modérée dans cinq cas (5%) et sévère dans deux cas (2%). Au niveau de l'antre, la MI retrouvée dans 48 cas (48%) était légère dans 37 cas (37%), modérée dans huit cas (8%) et sévère dans trois cas (3%). La MI antrale était en corrélation statistique très significative (p <0,001) avec la MI fundique. La répartition des stades de OLGA selon l'intensité de l'MI est résumée dans le Tableau 4. On notait une différence très significative entre les stades OLGA en fonction de l'intensité de la MI (p<0,005). Les gastrites chroniques à faible risque selon OLGA présentaient une MI dans seulement 29% des stades 0, et 50% des stades I avec une intensité le plus souvent légère. Nos six cas de gastrites à haut risque (stades III et IV), étaient associées à une MI d'intensité « modérée à sévère » dans quatre cas/six.

### Corrélation entre les classifications OLGA et OLGIM

En étudiant la liaison entre les différents stades des classifications OLGA et OLGIM, 47 cas (47%) de nos gastrites à *H pylori* étaient au même stade selon les deux classifications: 17 cas au stade 0, 26 cas au stade I, un cas au stade II, deux cas au stade III et un cas au stade IV (Tableau 5). Au total, 91 cas (91%) appartenaient aux stades à faible risque (0, I et II) selon les deux classifications et quatre cas (4%) étaient aux stades à haut risque (III et IV). Une corrélation positive et hautement significative (p<0,005) a été retrouvée entre OLGA et OLGIM (Tableau 3). Une discordance a été relevée dans cinq cas (5%) puisque deux cas (2%) classés à haut risque selon OLGA (stade III) étaient à bas risque selon OLGIM (un cas au stade 0 et un cas au stade I) et trois cas (3%) classés à haut risque selon OLGIM (stade III) étaient à bas risque selon OLGA (un cas stade 0 et deux cas stade I).

**Tableau 5:** Corrélation entre OLGA et OLGIM au cours des gastrites à *H pylori*

		Les stades OLGA					Total
		0	I	II	III	IV	
**Les stades OLGIM**	**0**	**17**	31	0	1	0	49
	**I**	6	**26**	7	1	0	40
	**II**	0	3	**1**	0	0	4
	**III**	1	2	0	**2**	0	5
	**IV**	0	0	0	1	**1**	2
**Total**		24	62	8	5	1	100

## Discussion

Les données épidémiologiques montrent une étroite corrélation entre la classification OLGA et l’âge. L’âge moyen des patients porteurs de gastrites chroniques à haut risque (III et IV de OLGA), est estimé entre 60 et 69 ans selon les séries et parait significativement supérieur à celui des patients atteints de gastrites chroniques de faible risque, estimé entre 43 à 65 ans [3–5]. Dans notre étude et en concordance avec les données de la littérature, une liaison significative est retrouvée (p<0,05) entre l’âge et la classification d'OLGA. Les données de la littérature montrent des variations significatives des moyennes d’âge en fonction des stades d'OLGIM; les patients classés aux stades à haut risque présentaient un âge moyen compris entre 62 et 72 ans, plus élevé que celui des patients des stades à faible risque [3, 4]. Dans notre étude, il n'a pas été relevé de corrélation entre la classification OLGIM et l’âge. Ceci serait très probablement lié au faible effectif. Le sexe n'a aucune influence sur la prévalence de la gastrite chronique à *H pylori*, aussi bien dans la littérature mondiale que dans notre travail [1, 4]. De même, qu'il n'a pas été retrouvé de corrélation entre le sexe et les deux classifications d'OLGA et OLGIM [4]. La densité de *H pylori* est variable selon les séries, estimée à « légère » dans 30%, « modérée » dans 34% et « sévère » dans 36% des cas [6]. Dans notre étude, et conformément aux données de la littérature, l’*H pylori* était retrouvé avec une densité « légère » dans 37%, modérée dans 26% et sévère 37%. Selon la classification de OLGA, les gastrites chroniques à faible risque étaient significativement associées à une infection à *H pylori* de forte densité, estimée à 71% pour le stade 0, 63% pour le stade I et 75% pour le stade II. L’*H pylori* est retrouvé dans les études menées sur les gastrites chroniques comme significativement prédominant dans les formes à haut risque selon OLGA atteignant 92% [7]. Par ailleurs, il n'a pas été retrouvé, dans la revue de littérature de données comparatives entre la densité de cette infection et les différents stades de OLGA. Les gastrites à haut risque selon OLGA, se caractérisent par une atrophie marquée de la muqueuse, qui entrainerait des variations environnementales avec une perturbation de l’écosystème devenant inadéquat pour la multiplication bactérienne. Les stades à faible risque représentent plus de 90% des gastrites chroniques avec une nette prédominance du stade 0 [7]. Conformément aux données de la littérature, notre série retrouve une nette prédominance des stades à faible risque (94%). Le risque évolutif de nos gastrites chroniques est semblable aux normes avec toutefois un taux plus faible de stade 0 (24%) et plus élevé de stade I (62%). Les gastrites classées aux stades à haut risque sont modérément ou sévèrement « atrophiques » s'observent à forte fréquence dans les régions à forte prévalence d’*H pylori* et de cancer gastrique. Les gastrites chroniques de haut risque évolutif représentent 25,8% en Corée [7]. Les stades à haut risque représentent 6,4% dans les séries italiennes avec une nette prédominance du stade III (44%) avec un très faible taux de stade IV (<1%) [4]. Conformément aux données de la littérature européenne, nos gastrites à *H pylori* à haut risque présentent dans 6% des cas, avec 5% de stade III et 1% de stade IV. Selon la classification de OLGIM, les gastrites chroniques à faible risque étaient significativement associées à une infection à *H pylori* de forte densité, estimée à 61% pour le stade 0, 68% pour le stade I et 100% pour le stade II. L’étude coréenne menée par Nam et al. [7] a montré que 90% des gastrites chroniques à haut risque selon OLGIM étaient d’étiologie infectieuse, sans en préciser la densité par rapport aux formes à faible risque. Ces constatations sont similaires aux données de l’étude comparative entre OLGA et *H pylori*; elles sont également expliquées par les modifications de l’écosystème devenant inapproprié pour la multiplication bactérienne dans les formes à haut risque d'OLGIM qui se caractérisent par une MI étendue. Conformément aux données de la littérature, notre série retrouve une nette prédominance des stades à faible risque. Le stade 0 selon OLGIM est le plus représenté. De même, nos gastrites à *H pylori* présentent selon OLGIM un haut risque évolutif dans 7% des cas avec une prédominance de stade III [7].

Dans notre étude, l'atrophie glandulaire s'observe dans 76% des cas, avec une prédominance antrale, et une intensité estimée à légère dans 62 cas des cas, modérée dans 13 cas (13%) et sévère dans un seul cas (1%). Selon Chen et al. [6], l'atrophie glandulaire est observée dans 52% des gastrites chroniques, avec une intensité légère dans 28% des cas, modérée dans 20% et sévère dans 4% des cas. L’étude de l'atrophie glandulaire selon la classification d'OLGIM, objective une corrélation franche entre l'atrophie glandulaire et les stades à haut risque [1]. En accord avec la littérature, notre étude a montré l’étroite corrélation entre l'atrophie et les stades à haut risque. Dans notre étude, la MI présentait une forte prévalence proche de celle relevée dans les séries asiatiques (à 49%) avec une prédominance antrale et une intensité légère dans 62 cas, modérée dans 6 cas et sévère dans trois cas [6]. Pour Capelle et al. [3], la MI est relevée dans 54% des gastrites chroniques à faible risque et dans 80% des formes à haut risque. Dans notre étude, et conformément aux données de la littérature, les gastrites chroniques à faible risque selon OLGA présentaient une MI dans seulement 29% des stades 0, et 50% des stades I. Nos six cas de gastrites à haut risque (stades III et IV), étaient associées à une MI d'intensité « modérée à sévère » dans quatre cas/six.

La classification d'OLGA, en complément au Sydney System, permet de répondre aux attentes des cliniciens en sélectionnant, sur des bases morphologiques, les formes de gastrites chroniques à haut risque qui nécessiteraient une surveillance particulière. En pratique, son application semble aisée avec toutefois certaines difficultés pour apprécier le degré d'atrophie soulevant des problèmes de reproductibilité inter-observateurs [3, 5]. Contrairement à la classification de OLGA, la classification de OLGIM a le mérite de pouvoir sélectionner les formes de gastrites chroniques à haut risque évolutif avec une reproductibilité inter-observateurs nettement meilleure (score kappa variable de 0,68 à 0,92 selon les séries) [3, 8]. Dans notre étude, et en accord avec la littérature, une corrélation positive et hautement significative (p<0,005) a été retrouvée entre OLGA et OLGIM. Une discordance a été relevée dans 5 cas (5%) : OLGIM sous-évaluait 40 cas parmi lesquels deux cas (2%) classés à haut risque selon OLGA (stade III) devenant à bas risque selon OLGIM et OLGA sous-évaluait 13 cas dont trois cas (3%) classés à haut risque selon OLGIM (stade III) considérés à bas risque selon OLGA. Cependant, plusieurs auteurs tout en reconnaissant les avantages de OLGIM, affirment que certains patients classés aux stades à haut risque selon OLGA sont sous évalués selon OLGIM et seraient par la suite privés de la surveillance adéquate. Ce groupe de patients est diversement évalué selon les séries; il variait de 0,003% à 3% des cas [1, 5, 7, 9], valeur très proche de celle retrouvée dans notre série (2%). OLGA et OLGIM représentent deux modèles de classification histopronostique ayant l'avantage de fournir aux cliniciens des informations précises et pertinentes permettant de sélectionner les formes nécessitant une surveillance rigoureuse. En appliquant les recommandations du Management of Precancerous conditions and lesions in the Stomach ou MAPS [10] qui proposent une surveillance endoscopique tous les trois ans pour les formes de gastrites à haut risque selon OLGA ou OLGIM, à notre étude, 91% de nos patients étaient classés aux stades à faible risque selon OLGA et OLGIM nécessitant un traitement éradicateur d’*H pylori* sans surveillance particulière. Dans 9% des cas, nos patients étaient classés à haut risque selon OLGA et OLGIM ce qui aurait imposé un traitement éradicateur d’*H pylori* avec une surveillance endoscopique tous les trois ans.

## Conclusion

A la lumière de cette étude et en accord aux données de la littérature, nous recommandons de point de vue pratique et afin d'adapter à nos patients la prise en charge et le suivi endoscopique proposés par les recommandations internationales du MAPS [10] d'appliquer d'une part, les classifications OLGA et OLGIM dans les gastrites chroniques à *H pylori* et, de préciser, sur le compte rendu anatomo-pathologique, le risque évolutif de la gastrite. Des études épidémiologiques prospectives et multicentriques seraient nécessaires pour valider les recommandations MAPS à notre population.

### Qu'est ce qui est connu sur ce sujet

- La gastrite chronique englobe est le plus souvent liée à *H pylori*.
- La gastrite à *H pylori* présente un risque de cancérisation en rapport avec l'atrophie et la métaplasie intestinale.
- Les classifications OLGA et OLGIM permettent d'individualiser les formes à haut risque évolutif.

### Qu'est-ce que votre étude apporte de nouveau

- OLGA et OLGIM représentent deux classification histopronostiques permettent de fournir aux cliniciens des informations précises et pertinentes en sélectionnant les formes nécessitant une surveillance rigoureuse.
- L'application des classifications OLGA et OLGIM dans les gastrites chroniques à *H pylori*.
- L'adaption aux patients la prise en charge et le suivi endoscopique proposés par les recommandations internationales du MAPS.
